# Low-Temperature Structural Study of Smectic C_A_* Glass by X‑ray Diffraction

**DOI:** 10.1021/acs.jpcb.5c03603

**Published:** 2025-06-14

**Authors:** Aleksandra Deptuch, Marcin Kozieł, Marcin Piwowarczyk, Magdalena Urbańska, Ewa Juszyńska-Gałązka

**Affiliations:** 1 Institute of Nuclear Physics Polish Academy of Sciences, Radzikowskiego 152, PL-31342 Kraków, Poland; 2 Faculty of Chemistry, Jagiellonian University, Gronostajowa 2, PL-30387 Kraków, Poland; 3 Institute of Chemistry, 69698 Military University of Technology, Kaliskiego 2, PL-00908 Warsaw, Poland; 4 Research Center for Thermal and Entropic Science, Graduate School of Science, Osaka University, 560-0043 Osaka, Japan

## Abstract

The liquid crystalline compound, forming the glass of the smectic C_A_* phase, is investigated by X-ray diffraction in the 18–298 K range. The characteristic distances within the smectic C_A_* phase are determined, and the specific volume is estimated. The electron density profile along the smectic layer normal is inferred and compared with the results of the density functional theory calculations. Observations of the selective reflection of the visible light investigate the helical ordering within the smectic C_A_* glass. The results indicate slow change with temperature of the smectic layer spacing, intermolecular distances, and electron density distribution below the glass transition temperature. The change in the temperature dependence of the specific volume is well below the glass transition temperature. Meanwhile, the relative range of the short-range order within the smectic layers and the helix pitch are rather constant in the glassy state.

## Graphical abstract

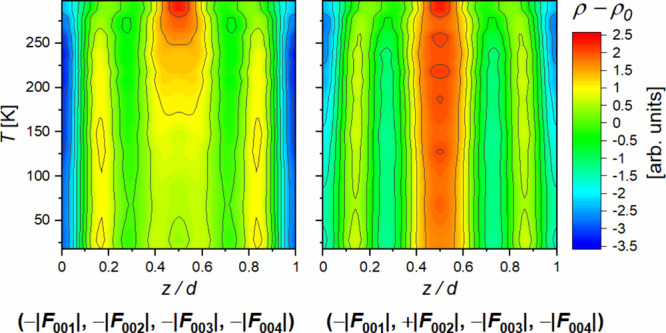

## 1 Introduction

Smectic phases are a type of thermotropic liquid crystalline phases characterized by the lamellar positional order of molecules.^1–4^ In some smectic phases, the switching in the electric field (ferro-, ferri-, or antiferroelectricity) is observed. Therefore, the smectogenic compounds are investigated for their potential application in liquid crystal displays.^5–9^ Fluorinated liquid crystalline compounds abbreviated as 3F*m*X_1_PhX_2_ *n* (Figure 1) were also synthesized initially for this purpose. The 3F*m*X_1_PhX_2_ *n* compounds form the smectic C* phase with ferroelectric properties and the smectic C_A_* phase with antiferroelectric properties in the surface-stabilized geometry; many of them exhibit both phases: SmC* at higher temperatures and SmC_A_* at lower temperatures.^10–14^ The SmC* and SmC_A_* phases show, respectively, the locally synclinic and anticlinic orders of the tilt angle of chiral molecules within layers.^5^ On the scale of hundreds of nanometers, the azimuth of the molecular tilt changes helically, and the homeotropically oriented sample exhibits selective reflection in the ultraviolet, visible, or near-infrared range.^10,13^ The helix axis is perpendicular to the smectic layers. Consequently, the effect related to the changes in the helix pitch is visible for a sample in the homeotropic alignment, with the smectic layers parallel to the sample’s plane.^5^

**Figure 1:**
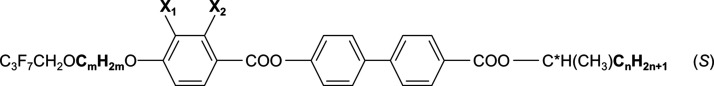
General molecular formula of 3F*m*X_1_PhX_2_ *n*. In this work, *m* = 5, X_1_ = H, X_2_ = F, *n* = 6.

Later studies prove that some 3F*m*X_1_PhX_2_ *n* compounds show very good glassforming properties,^14–16^ which makes them suitable compounds for the basic studies of the glass transition in partially ordered phases. Although there are many reports of the smectogenic small-molecule (nonpolymeric) glassformers, to give as examples refs 14–23 , the structural studies deep in the glassy smectic state are scarcer.^14,24–28^ Based on a literature review, the lowest reported temperature for X-ray diffraction data on the smectic glass is 83–85 K.^26–28^

The 3F5HPhF6 compound, investigated herein, has a low melting temperature of the crystal phase, equal to 301.3 K.^10^ It is a good glassformer, where the crystallization on cooling is not observed, and the glass of the SmC_A_* phase is formed.^14^ This study presents the X-ray diffraction data obtained for the supercooled and vitrified SmC_A_* phase of 3F5HPhF6 down to 18 K. The smectic layer spacing, intermolecular distance, and correlation length of the intralayer order are determined. The density and specific volume are estimated from the characteristic distances. Three variants of the distribution of the electron density along the smectic layer normal are inferred from the integrated intensities of the diffraction peaks from the smectic layers and compared with the electron density distribution estimated from the results of the density functional theory calculations. Additionally, the selective reflection of the visible light is investigated down to 173 K to determine if the helical pitch changes below the glass transition temperature.

## 2 Methods

(*S*)-4′-(1-Methylheptyloxycarbonyl)­biphenyl-4-yl 4-[5-(2,2,3,3,4,4,4-heptafluorobutoxy)­pentyl-1-oxy]-2-fluorobenzoate, denoted as 3F5HPhF6, was synthesized according to the route presented in ref 10.

Differential scanning calorimetry (DSC) measurements were performed using a TA Instruments DSC 2500 calorimeter for a sample weighing 7.17 mg in a hermetic aluminum pan. Thermograms were registered during cooling and heating at the 5, 10, and 20 K/min rates between 173 and 403 K. DSC data analysis was done in TRIOS.

Polarizing optical microscopy (POM) measurements in transmission mode were carried out using a Leica DM2700 P microscope with the Linkam temperature attachment for a sample placed between two thin glass slides without the aligning layer. Images were taken every 6 s during cooling and heating at 10 K/min in the 188–393 K range. Selective reflection was investigated in reflection mode using the same equipment for a sample placed within the electro-optic cell WAT-3A with a thickness of 5 μm and the polymer layer providing the homeotropic alignment. Images were taken every 5 s during cooling and subsequent heating at 10 K/min in the 173–377 K range and 1 K/min in the 367–377 K range. The weighted average luminance of textures was calculated in TOApy,^29^ and the average contributions of the red, green, and blue components were calculated in ImageJ.^30^

X-ray diffraction (XRD) measurements were performed using a Malvern Panalytical Empyrean 3 diffractometer equipped with an Oxford Cryosystems Phenix cryostat (Bragg–Brentano geometry, Cu Kα radiation, λ = 1.540562 Å,^31^ measurement over the range of 2θ = 2–40°). The sample was initially heated to 313 K to melt the crystal phase. Then, the XRD patterns were registered on cooling in selected temperatures from 298 to 18 K and upon subsequent heating to 298 K. XRD data analysis was done in WinPLOTR^32^ and OriginPro.

Density functional theory (DFT) calculations were done in Gaussian 16 ^33^ for an isolated 3F5HPhF6 molecule with the B3LYP-D3­(BJ) exchange-correlation functional^34–37^ and def2TZVPP basis set.^38^ The starting model was prepared in Avogadro,^39^ and the optimized model was visualized in VESTA.^40^

## 3 Results and Discussion

### 3.1 Differential Scanning Calorimetry

The DSC thermograms of 3F5HPhF6 (Figure 2) show a peak with the onset temperature^41^ at 372.2 K on cooling and 371.4 K on heating and the average enthalpy change of 6.2 kJ/mol. This peak corresponds to Iso/SmA*/SmC*/SmC_A_* transitions, where Iso is the isotropic liquid. Only at the 5 K/min rate is the minor anomaly corresponding to the SmC*/SmC_A_* transition visible, with the onset temperature of 369.3 K on cooling and 370.8 K on heating and the average enthalpy change less than 0.1 kJ/mol (inset in Figure 2). The earlier XRD results at high temperatures^42^ show that the SmA* phase of 3F5HPhF6, present only in a very narrow temperature range, is the de Vries phase, i.e., the SmA*/SmC* transition occurs with a negligible change in the smectic layer spacing. Such a continuous transition is not visible in the obtained DSC thermogram. The glass transition temperature of the SmC_A_* phase, determined at the half-height of the step in the heat capacity,^43^ is equal to 227 K on cooling and 231 K on heating, with the average step in the heat capacity of 0.17 kJ/(mol·K). Noteworthily, in the earlier DSC results reported in ref 14, the step in the heat capacity was not visible on cooling due to a bent baseline, and *T*_g_ was estimated from the kink in the DSC thermogram, leading to lower values. The *T*_g_ values obtained in this study at the half-height of the step in the heat flow are in better agreement with *T*_g_ = 229–230 K determined from the α-relaxation time in the dielectric spectra of 3F5HPhF6.^15^

**Figure 2:**
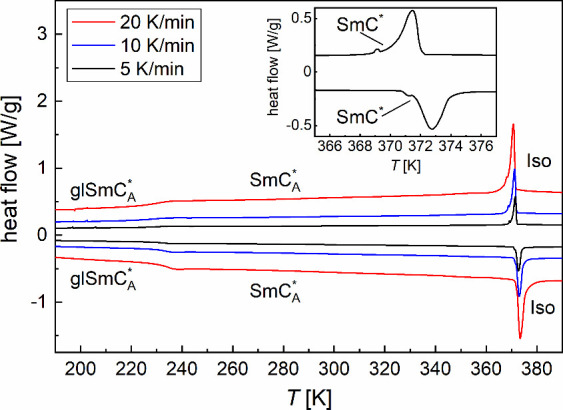
DSC thermograms of 3F5HPhF6. The inset shows the close-up of the SmC*/SmC_A_* transition for the 5 K/min rate.

### 3.2 Polarizing Optical Microscopy and Selective Reflection of Visible Light

The representative POM textures of 3F5HPhF6, registered in the transmission mode, and the corresponding luminance vs temperature plots are shown in Figures S1–S4 in the Supporting Information (SM). The SmC* phase shows a green texture, while POM does not detect the SmA* phase. Despite the absence of the aligning layer, the alignment of the sample is mainly homeotropic because the helix inversion (the increase of the pitch, unwinding of the helix, and subsequent decrease of the pitch with temperature) within the SmC_A_* phase^10^ is observed as a significant change in luminance. The helix inversion temperature shows some hysteresis, occurring at ca. 325 K on cooling and 337 K on heating. The glass transition leads to a minor, step-like increase in luminance. The *T*_g_ values estimated from the POM observations are equal to ca. 240 K on cooling and 245 K on heating, higher than those obtained by DSC. In the heating run, the disordered texture arises above *T*_g_; see the texture registered at 290 K in Figure S3. The DSC results do not indicate the cold crystallization. Thus, such POM observation is explained by the nucleation, which is not followed by the crystal growth. The appearance of very small crystallites can disturb the alignment in the SmC_A_* phase, but it will not be visible in the DSC scan. The texture returns to the homeotropic alignment at ca. 310 K, about 10 K above the melting temperature of the crystal phase reported in ref 10.

The images obtained in the reflection mode on cooling at 10 K/min and the numerical analysis results are presented in Figures 3 and 4. The corresponding results for heating at 10 K/min are shown in Figures S5 and S6 in SM. The reflection of the green light is observed for the SmC* phase (for more detailed observations within the SmC* phase, performed at the 1 K/min cooling/heating rate, see Figures S7 and S8 in SM). In the SmC_A_* phase, the dark texture is observed. The helix inversion at 314 K on cooling and 345 K on heating shows a peak in the luminance. The hysteresis in the helix inversion temperature is wider than for the sample between glass slides without aligning layers. The helix pitch values presented in ref 10 down to 283 K imply that the selective reflection of the red light should be observed at lower temperatures. Indeed, the red color arises in the images of the SmC_A_* phase below ca. 280 K and remains in the SmC_A_* glass; see the representative images registered in 260 and 173 K in Figure 3. The numerical analysis (Figure 4) shows that the contribution of the red component increases on cooling from 280 to 240 K. In comparison, below 240 K, all components are constant, which is attributed to the glass transition. It indicates that the helix pitch does not change noticeably with temperature in the vitrified state. On subsequent heating, an additional peak in the luminance is observed at 270–280 K, which is explained by nucleation. Due to the presence of the aligning layer, the sample returns to the previous alignment at ∼300 K, corresponding to the crystal phase’s melting temperature.^10^

**Figure 3:**
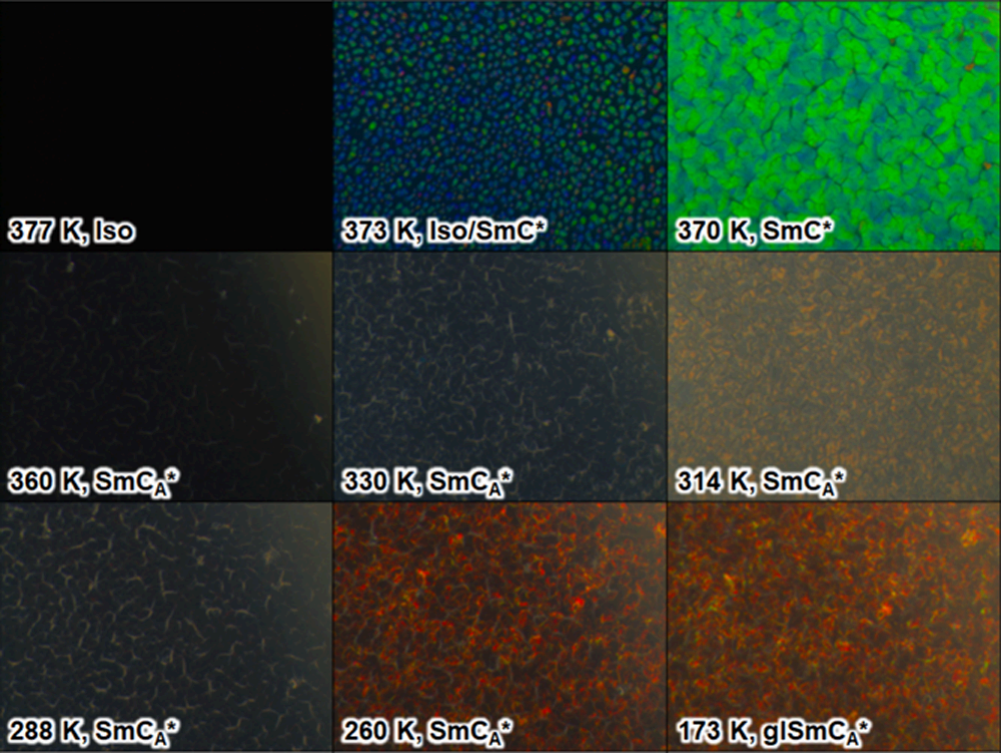
POM textures of 3F5HPhF6 registered on cooling at 10 K/min in the reflection mode. Each image shows an area of 622 × 466 μm^2^.

**Figure 4:**
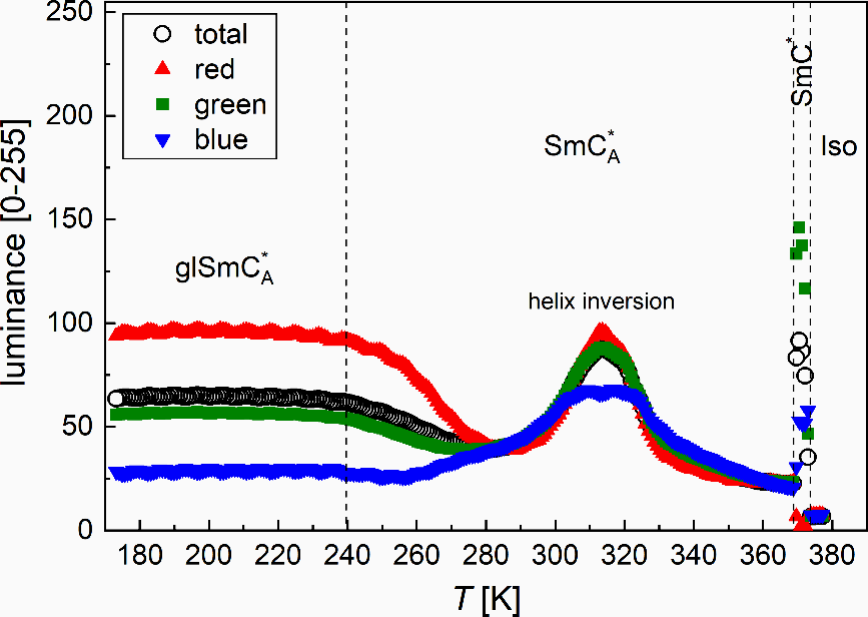
Average weighted luminance and separate contributions of the red, green, and blue components of the POM textures of 3F5HPhF6 obtained on cooling at 10 K/min in the reflection mode.

The wavelength of selectively reflected light λ is equal to the helix pitch *p* multiplied by the average refractive index *n*_av_: λ = *n*_av_ *p*.^44^ The temperature dependence of the inverse helix pitch, 1/*p*, can be described by a linear function.^44^ It enables extrapolation of λ to low temperatures by fitting a function λ­(*T*) = λ_0_/(1 + *DT*) to λ values in the 283–308 K range (Figure 5), obtained from the helix pitch from ref 10 and refraction index *n*_av_ = 1.5,^45^ also assumed in ref 10. The spectral range of visible light is between 360–400 and 760–830 nm.^46^ According to the extrapolation in Figure 5, selectively reflected light enters the visible range (830 nm) at ∼275 K and leaves the visible range (360 nm) at ∼210 K. The λ value expected at 240 K, according to this extrapolation, is equal to 485 nm, which is well below the range of the red light.^46^ However, in microscopic observations, only red images are recorded at 173–280 K. It indicates that the helix pitch decreases more slowly with decreasing temperature on approaching *T*_g_ than at higher temperatures and is approximately constant below *T*_g_.

**Figure 5:**
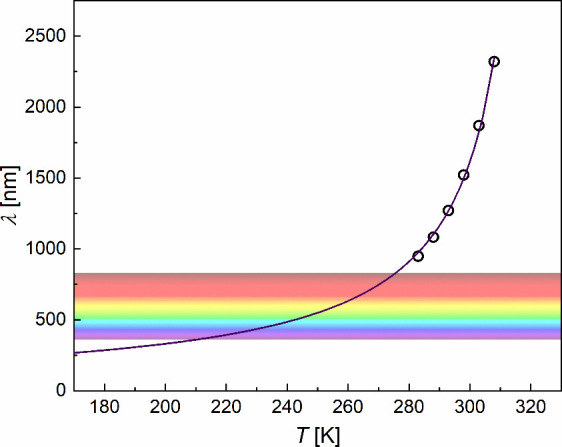
Wavelength of selectively reflected light vs temperature estimated from the helical pitch at 283–308 K, adapted from ref 10 with permission from the Royal Society of Chemistry. The color bar shows schematically the visible-light range.

### 3.3 X-ray Diffraction and Molecular Modeling

The XRD patterns of 3F5HPhF6 and the empty sample holder are shown in Figure 6. The sharp peak at 2θ ≈ 2.5° and broad maxima at 2θ ≈ 4.2 and 6.7° originate from the sample holder. The sharp peak at 2θ ≈ 2.9° and its higher harmonics, up to the fourth one, originate from the quasi-long-range positional order in the SmC_A_* phase, i.e., the smectic layers.^1,2^ The broad maximum at 2θ ≈ 19–20° is related to the short-range positional order within the smectic layers.^47^ In the heating run, numerous sharp peaks at 298 K indicate partial cold crystallization. The appearance of very small crystallites at lower temperatures, implied by the POM observations, is not visible in the XRD patterns. The Bragg equation^48^ relates the positions θ_*l*_ of the (00 *l*) peaks, where *l* = 1, 2, 3, 4, to the smectic layer spacing *d*:

$$\theta_{l}=\theta_{0}+arc\sin\left(\frac{l\lambda }{2d}\right)$$ 1

where θ_0_ is the systematic shift in the peak positions and λ = 1.540562 Å (Cu Kα1 characteristic wavelength^31^). The layer spacing was determined by fitting eq 1 to the experimental peak positions. At temperatures 288 and 298 K, only (001) and (003) peaks were visible; thus, the θ_0_ shift was taken from 278 K. The obtained results (Figure 7a) show that the layer spacing has a local maximum at 238 K, interpreted as a sign of the glass transition. In the glassy SmC_A_* phase, the layer spacing decreases on cooling, with a relative decrease of 2.0% from 238 to 18 K.

**Figure 6:**
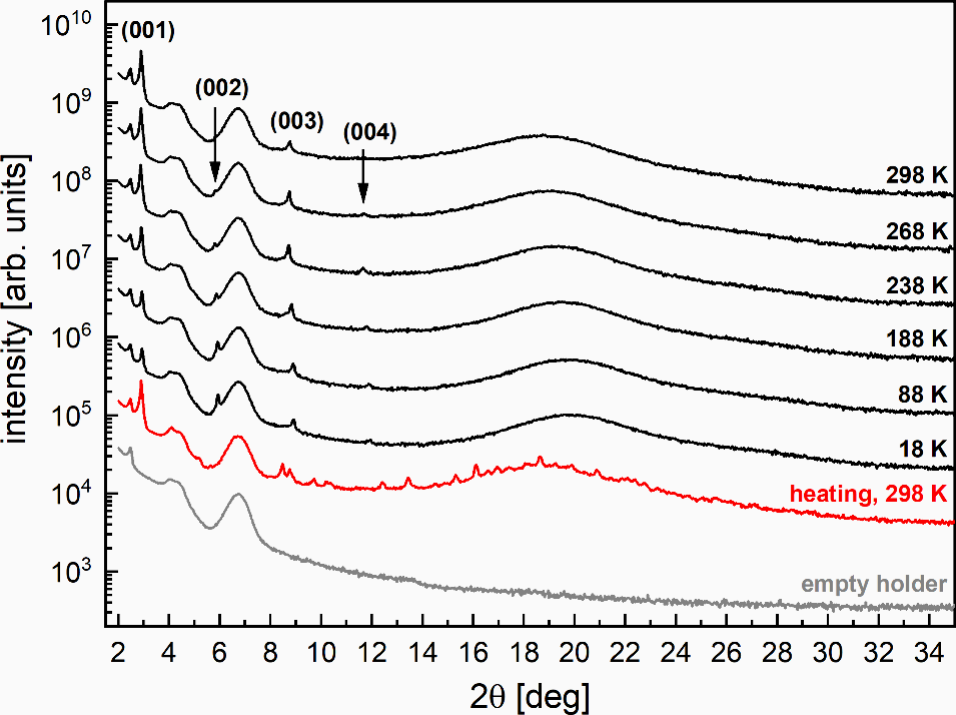
Selected XRD patterns of 3F5HPhF6 collected on cooling from 298 to 18 K and after gradual heating to 298 K. The bottom pattern was collected at room temperature for an empty holder. The sharp peaks arising from the smectic layer order are indicated.

**Figure 7:**
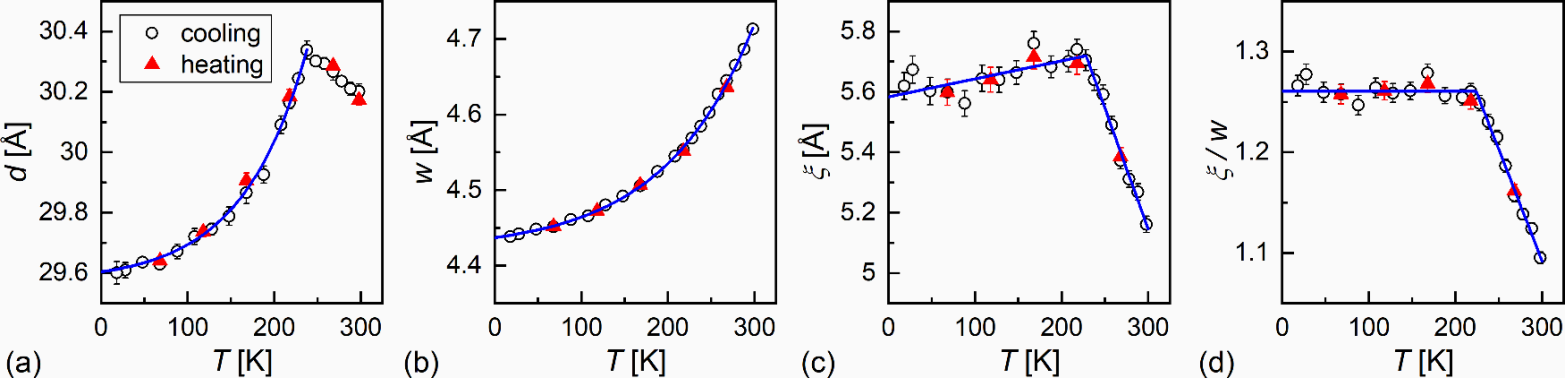
Smectic layer spacing (a), average intermolecular distance within the smectic layers (b), correlation length of the short-range order (c), and ratio of the correlation length and intermolecular distance (d) as a function of temperature in the supercooled and vitrified SmC_A_* phase of 3F5HPhF6. The legend in (a) is common for all panels.

The short-range order in the smectic layers is described by the correlation length ξ and the average distance *w* between molecules. After recalculation of the 2θ angle into the scattering vector *q* = 4π sin θ/λ, the wide maximum at 2θ ≈ 19–20° (*q*_0_ ≈ 1.3–1.4) is described by the Lorentz peak function:^47^

$$I\left(q\right)=\frac{A}{1+\xi^{2}{\left(q-q_{0}\right)}^{2}}+Bq+C$$ 2

where *A* is the peak height and *B* and *C* are the parameters of the linear background. The average distance, obtained as *w* = 2π/*q*_0_, decreases on cooling, with a relative decrease of 5.9% from 298 to 18 K (Figure 7b). The glass transition temperature cannot be inferred from the *w*(*T*) plot, as neither extrema nor discontinuities are observed at *T*_g_. The correlation length increases when cooling down to 218 K and shows a decreasing trend when cooling below 218 K (Figure 7c). The slight decrease of ξ in the glassy SmC_A_* state is caused by a simultaneous decrease in *w*. In the plot of the ξ/*w* ratio, one can see that above 218 K, the short-range positional order increases on cooling, while it practically does not change below 218 K (Figure 7d). Both *d* below *T*_g_ and *w* in the whole investigated range can be fitted empirically with an exponential function *y*(*T*) = *y*_0_ + *a* exp (*T*/*T*_0_), and their values extrapolated to 0 K (*y*_0_ + *a*) are equal to *d*_0_ = 29.60(2) Å and *w*_0_ = 4.437(3) Å. The ξ and ξ/*w* values can be fitted with two linear functions, with the intersection points at 228(4) and 224(3) K, respectively. The slope of ξ/*w* below *T*_g_ was fixed to zero. The average value of the ξ/*w* in the glassy SmC_A_* phase is 1.261(2) and corresponds to the nearest-neighbor correlations only.

The integrated intensity of the (001) peak from the smectic layers decreases on cooling (Figure 8a). The decrease in intensity accelerates below 250 K, which is around the glass transition region, and slows down below 88 K. The intensity of the (002) peak increases on cooling down to 68 K and slightly increases on further cooling. The intensity of the (003) peak has a broad maximum centered at 218 K, while the intensity of the (004) peak is approximately constant. The (001) peak is significantly stronger than the higher-order peaks, caused by the Lorentz-polarization factors *Lp*, strengthening the intensities at low 2θ angles. The united form of the *Lp* factors for the integrated intensities from the polycrystalline samples is^49–51^

$$Lp=\frac{1+\cos^{2}\left(2\theta \right)}{\sin^{2}\theta \cos\theta }$$ 3

**Figure 8:**
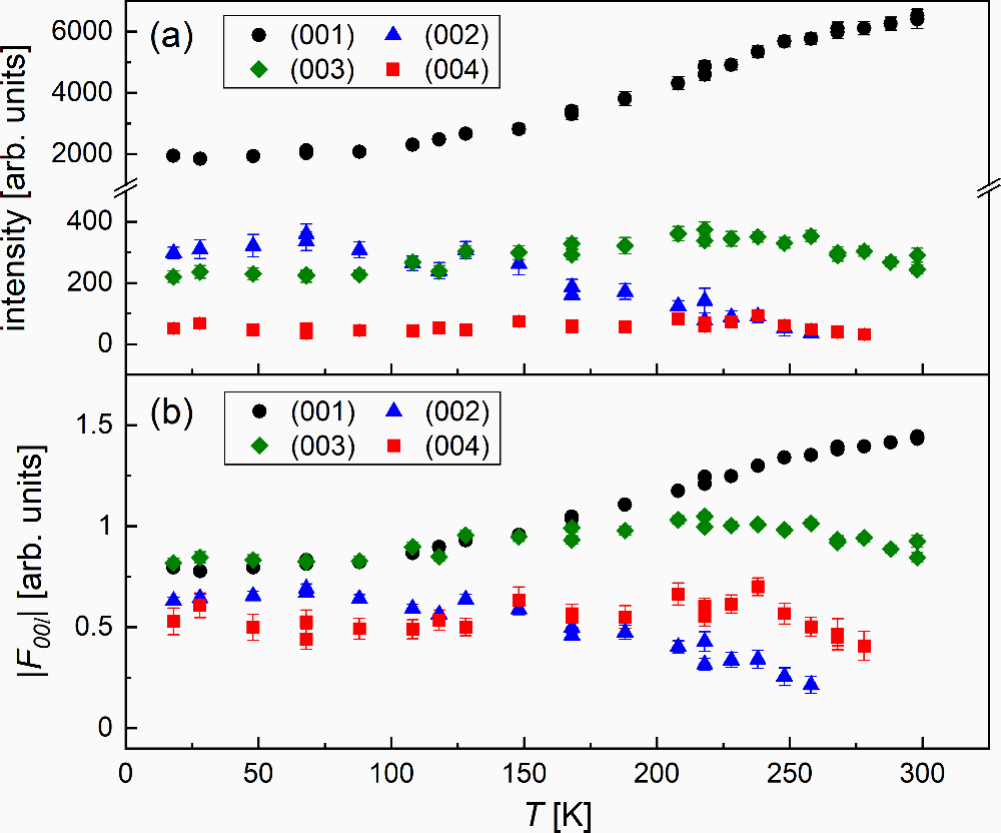
Integrated intensities of the (00 *l*) peaks from the smectic layers of supercooled and vitrified 3F5HPhF6 (a) and the absolute values of the structure factors, calculated from these intensities after correction by the Lorentz-polarization factors (b).

The *Lp*-corrected intensities are calculated as *I*_corr_ = *I*/(*Lp*). The relative *Lp*-corrected intensities of the (00 *l*) peaks depend on the distribution of the electron density ρ­(*z*) along the smectic layer normal.^1–3^ The molecules in the smectic phases can rotate around their short axes.^52,53^ Thus, it can be assumed that the ρ­(*z*) distribution is centrosymmetric in relation to the middle of the smectic layer:^54,55^

$$\rho \left(z\right)=\rho_{0}+\overset{4}{\underset{l=1}{\sum }}F_{00l}\cos\left(2\pi lz/d\right)$$ 4

where *F*_00*l*_ is the structure factor. Generally, structure factors are complex numbers and the ρ­(*z*) function is described by complex exp­(2π*ilz*/*d*) instead of cosines. The absolute *F*_00*l*_ values are determined as $|F_{00l}|=\sqrt{I_{\mathrm{corr}}}$ (Figure 8b), while their phases remain unknown. However, for the centrosymmetric ρ­(*z*) distribution, the simpler ([Disp-formula eq4]) formula can be applied, where *F*_00*l*_ are real numbers, and the phase problem is reduced to the choice of either (+) or (−) sign.^3,48,54^ With four diffraction peaks, it leads to 2^4^ = 16 combinations. Two sets with all opposite signs, for example (+,+,+,+) and (−,–,–,−), describe the same ρ­(*z*) distribution, only shifted by *d*/2.^54^ Since ρ­(*z*) has a period of *d*, it reduces the number of combinations to 8. Herein, we assume that a single smectic layer is located between *z* = 0 and 1; thus, the minimum of ρ­(*z*) is expected at *z* = 0. In this case, the *F*_001_ factor should be negative (−).

In the SmC_A_* phase at 298 K, there are two peaks, (001) and (003). The choice of the (−) sign of *F*_003_ retains the minimum of ρ­(*z*) at *z* = 0, while the choice of the (+) sign leads to a local maximum of ρ­(*z*) at *z* = 0, which is against our earlier assumption. The signs of *F*_002_ and *F*_004_ are less apparent. The analysis for the results at lower temperatures shows that only the (−,+,–,+) combination has to be excluded because it gives the minima in ρ­(*z*) close to the middle of the smectic layer, while the (−,–,–,−), (−,+,–,−), and (−,–,–,+) combinations describe ρ­(*z*) with minima at *z* = 0 (Figure 9).

**Figure 9:**
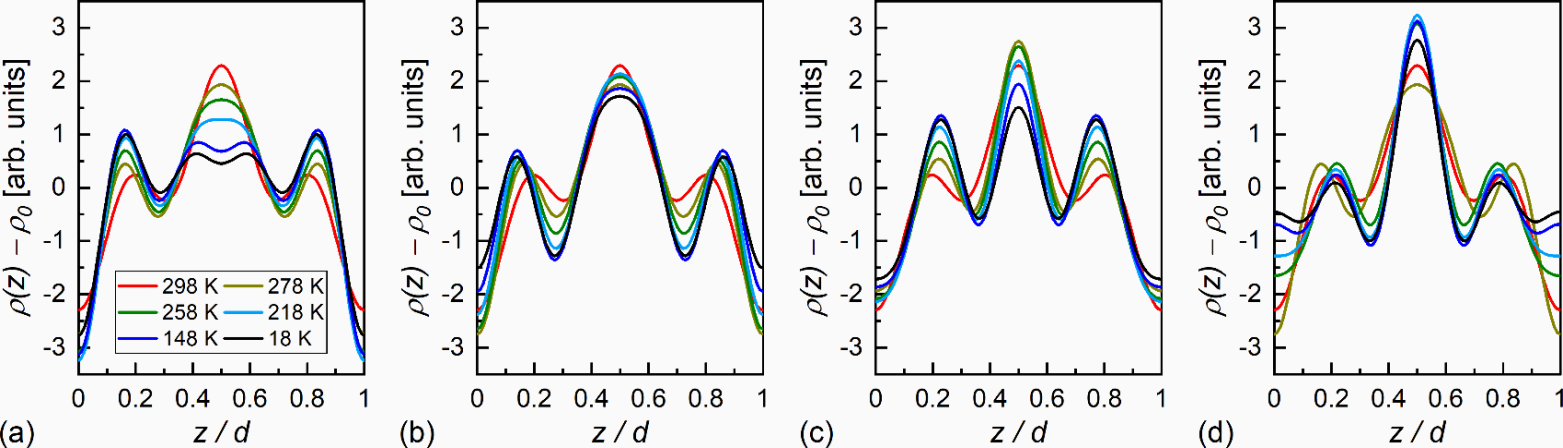
Electron density distribution along the smectic layer normal in 3F5HPhF6, calculated for various choices of the *F*_001_, *F*_002_, *F*_003_, and *F*_004_ signs: (−,–,–,−) (a), (−,+,–,−) (b), (−,–,–,+) (c), and (−,+,–,+) (d). The legend in (a) is common for all panels.

The obtained |*F*_00*l*_| values indicate that the electron density distribution along the layer normal evolves also below the glass transition temperature (Figure 7b). Above 168 K, the |*F*_001_| factor is dominant, and below 168 K, it decreases and becomes practically equal to |*F*_003_|. |*F*_002_| and |*F*_004_| are smaller than |*F*_001_| and |*F*_003_| in the whole temperature range. Down to 148 K, |*F*_002_| is smaller than |*F*_004_|, and below 148 K, this relationship reverses. Regardless of the choice of the *F*_002_ and *F*_004_ signs, ρ­(*z*) deviates strongly from the simple cosine function.

The electron density distribution can be approximated based on the DFT calculations.^56,57^ Figure 10 shows the 3F5HPhF6 molecular model. The single-crystal XRD results for the MHPOBC compound in a crystal phase^58,59^ as well as the recently published semiempirical and molecular dynamics calculations for the 3F5HPhF9 compound^13^ show that the C_*n*_H_2*n*_– chain forms an angle of ∼90° with the molecular core. Such conformation was assumed for the 3F5HPhF6 molecule. The bending in the −OCH_2_C_3_F_7_ part was included to match the experimental smectic layer spacing and the tilt angle of the molecular core equal to ca. 45°.^10,14^ If one includes the 1.09 and 1.44 Å radii for the H and F terminal atoms,^60^ the layer spacing equals 29.06 Å.

**Figure 10:**
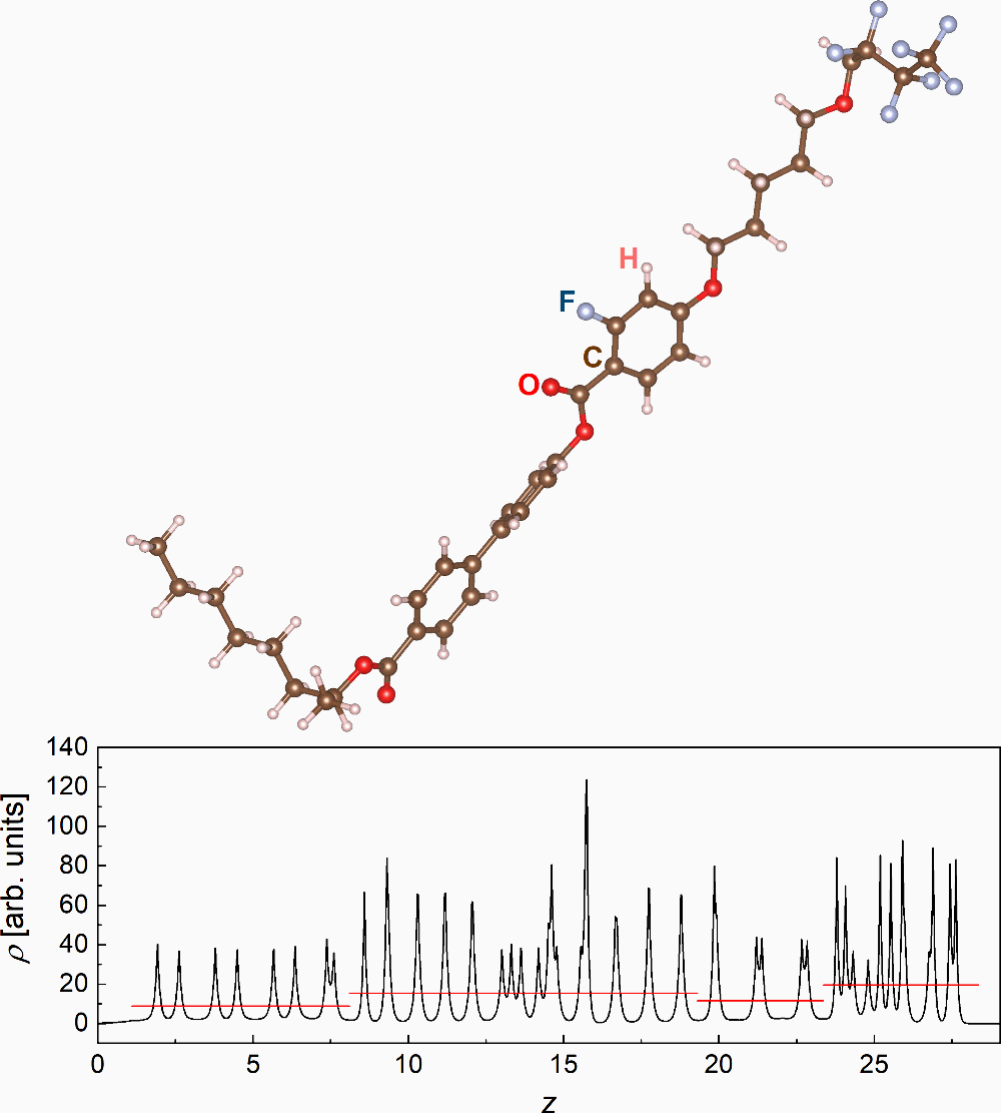
Model of the 3F5HPhF6 molecule optimized by the DFT method (B3LYP-D3­(BJ) functional, def2TZVPP basis set) and the corresponding electron density profile projected on the smectic layer normal, oriented in the horizontal direction. The red lines indicate the average electron density for various molecular parts.

The distribution of electron density around each atom was approximated by the cusp-like function *Z* ^2^ exp (−2*Z* | *z* – *z*_0_ |), based on the one mentioned in ref 61, where *Z* is the atomic number corrected by the Mulliken partial charge calculated by DFT for each atom and the pre-exponential parameter *Z* ^2^ provides the area of the cusp equal to *Z*. The average electron density, calculated separately for the C_6_H_13_–, −COOPhPhCOOPh–, −OC_5_H_10_–, and −OCH_2_C_3_F_7_ parts, is the highest for the fluorinated part of the achiral chain and the aromatic core.

Figure 11 presents the centrosymmetric electron density distribution, corresponding to the average over two 3F5HPhF6 molecules rotated by 180° with respect to the middle of the smectic layer. Equation 4 was fitted to obtain a smoother distribution. The obtained result resembles the ρ­(*z*) distributions determined from the XRD patterns for the *F*_001_, *F*_002_, *F*_003_, and *F*_004_ signs (−,–,–,−) (Figure 9a) and (−,+,–,−) (Figure 9b). The variant with the (−,–,–,+) choice of signs can be excluded at this stage, as it indicates the lateral maxima in ρ­(*z*) too close to the middle of the smectic layer. The DFT results show that these maxima originate from the fluorinated end of the achiral chain.

**Figure 11:**
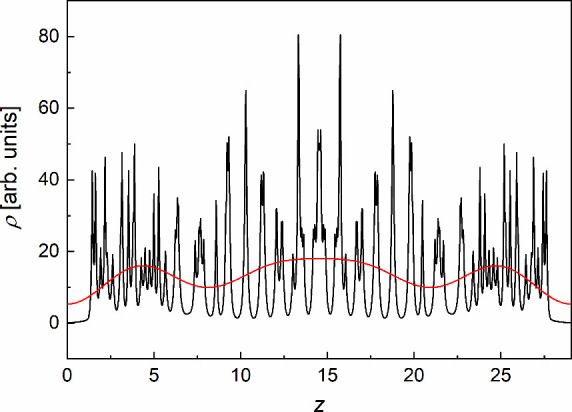
Centrosymmetric electron density distribution of 3F5HPhF6 based on the DFT calculations (B3LYP-D3­(BJ), def2TZVPP) along the smectic layer normal. The red line is the fitting result of eq 4.

At this point, only the (−,–,–,−) and (−,+,–,−) variants are left. The fitting result in Figure 11 corresponds to the (−,+,–,−) variant. However, the obtained *F*_002_ value is close to zero. Therefore, this result cannot be taken for granted, especially since it was performed mainly as a smoothing procedure. Nevertheless, the (−,–,–,−) and (−,+,–,−) variants lead to similar conclusions (Figure 9a,b): as the temperature decreases, the lateral maxima in ρ­(*z*) shift toward the borders of the smectic layer and the central maximum lowers. The (−,–,–,−) variant indicates additionally a slight split of the central maximum deep in the glassy state.

The distances *d* and *w* are applied to estimate the density of 3F5HPhF6 in the SmC_A_* phase. With an approximation of the hexagonal order of molecules, the volume per one molecule is calculated as $V=2dw^{2}/\sqrt{3}\approx 1.155dw^{2}$ .^62^ The molar mass of 3F5HPhF6 is 732.7 g/mol. The obtained density ρ is equal to 1.57 g/cm^3^ at 298 K and increases to 1.81 g/cm^3^ at 18 K. Figure 12 shows the specific volume *v* = 1/ρ vs temperature. The specific volume is expected to decrease approximately linearly with decreasing temperature, with a larger slope above *T*_g_ and a smaller slope below *T*_g_.^63–65^ However, the crossover temperature *T*_cross_ ≈ 168 K obtained for 3F5HPhF6 is well below calorimetric *T*_g_ obtained by DSC. Reference 65 mentions that *T*_g_ obtained from the specific volume can be lower than calorimetric *T*_g_ due to faster cooling in the DSC measurement. This explanation does not apply to 3F5HPhF6 because *T*_g_ = 229–230 K determined from dielectric spectra^15^ and *T*_g_ = 224–238 K obtained from the *d*, ξ, and ξ/*w* ratio indicate the same temperature region of the glass transition as calorimetric *T*_g_ = 227 K and *T*_g_ ≈ 240 K from POM observations. The difference between *T*_cross_ and *T*_g_ may be caused by orientational and partially positional order in the SmC_A_* phase. Instead of identifying *T*_cross_ as *T*_g_, one can relate *T*_cross_ to structural changes within the SmC_A_* glass. Figure 8b shows that the relationship between the main structure factors |*F*_001_| and |*F*_003_| is |*F*_001_| > | *F*_003_| above *T*_cross_ ≈ 168 K and |*F*_001_| ≈ | *F*_003_| below *T*_cross_. Moreover, the relaxation time of the secondary β-process in the SmC_A_* glass, investigated for 3F5HPhF6 in ref 15, reaches 100 s at ∼170 K, as extrapolated by fitting the Arrhenius formula. The β-process, interpreted as rotations of aromatic rings within the 3F5HPhF6 molecules,^15^ significantly slows down below *T*_cross_. This, consequently, modifies possibly both the electron density profile along the smectic layer normal and thermal expansion coefficients.

**Figure 12:**
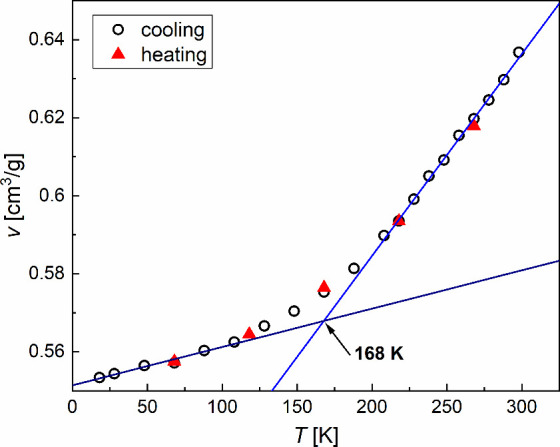
Specific volume of 3F5HPhF6, estimated from XRD patterns, as a function of temperature. The specific volume extrapolated to 0 K is equal to 0.5514(6) cm^3^/g, which corresponds to the density 1.819(2) g/cm^3^.

## 4 Conclusions

The structural studies of the vitrified SmC_A_* phase in the 3F5HPhF6 compound were carried out using the XRD method and DFT calculations. The smectic layer spacing shows a local maximum in the glass transition region. In contrast, the average distance between molecules within layers decreases on cooling without any anomalies around the glass transition temperature. The correlation length of the short-range positional order within layers remains constant in the vitrified state with respect to the average intermolecular distance.

The electron density profile is assumed to be centrosymmetric because the molecules can rotate around their short axes. The intensities of diffraction peaks from the smectic layer order show the comparable contribution of the cos­(2π*z*/*d*) and cos­(6π*z*/*d*) components to the electron density profile along the smectic layer normal, which results in three local maxima, one in the center and two close to the borders of the smectic layer. The central maximum corresponds to the fluorinated molecular core, and the lateral maxima correspond to the fluorinated end of the achiral chain. The *F*_001_, *F*_003_, and *F*_004_ structure factors are determined to be negative because only this combination provides the electron density distribution in agreement with the DFT calculations. The sign of the *F*_002_ factor remains ambiguous. Despite this, both the (−,–,–,−) and (−,+,–,−) variants indicate the shift of the fluorinated chain ends toward the borders of the smectic layers with decreasing temperature.

The linear dependences of the specific volume at low and high temperatures show the crossover temperature *T*_cross_ ≈ 168 K well below the glass transition temperature. The crossover temperature corresponds to significant slowing down of the secondary β-process and equalization of the *F*_001_ and *F*_003_ structure factors.

The microscopic observations of the selective reflection show that the helix pitch in the SmC_A_* phase is practically constant below the glass transition temperature. It can be assumed that any undetected, small changes in the pitch might be the effect of the shrinking in the smectic layer spacing, as the helix axis is perpendicular to the smectic layers (along the smectic layer normal).

The presented results show that the glass transition affects the properties of the SmC_A_* phase to various extents. Also, the determined glass transition temperature may differ depending on the investigated property. The dynamic *T*_g_ corresponding to the α-relaxation time equal to 100 s is equal to 229–230 K;^15^ the calorimetric *T*_g_ determined from the middle in the step of the heat capacity is equal to 227–231 K; and the temperature dependence of the intralayer short-range order indicates *T*_g_ = 224–228 K. Meanwhile, the smectic layer spacing has a local maximum at *T*_g_ = 238 K and the observations of selective reflection, as well as the POM measurements, indicate *T*_g_ = 240 K. It suggests that the structure along the smectic layer normal—layer spacing at the molecular scale and helix pitch at the mesoscopic scale—are affected by the glass transition at higher temperatures, at least for 3F5HPhF6.

## Supplementary Material



## Notes

Additional POM textures obtained in transmission and reflection modes together with the numerical analysis (PDF)
