# Supplementary material for: Low-Temperature Structural Study of Smectic CA* Glass by X‑ray Diffraction
Source: J Phys Chem B. 2025 Jun 14;129(25):6455–63. doi: 10.1021/acs.jpcb.5c03603 (PMC12207577; doi:10.1021/acs.jpcb.5c03603)
Supplement: Supplementary file 1 [file jp5c03603_si_001.pdf]

Low-temperature structural study of smectic C_A* glass by X-ray diffraction

Aleksandra Deptuch[1,*], Marcin Kozieł[2], Marcin Piwowarczyk[1], Magdalena Urbańska[3], Ewa Juszyńska-Gałązka[1,4]

[1] Institute of Nuclear Physics Polish Academy of Sciences, Radzikowskiego 152, PL-31342 Kraków, Poland

[2] Faculty of Chemistry, Jagiellonian University, Gronostajowa 2, PL-30387, Kraków, Poland

[3] Institute of Chemistry, Military University of Technology, Kaliskiego 2, PL-00908 Warsaw, Poland

[4] Research Center for Thermal and Entropic Science, Graduate School of Science, Osaka University, 560-0043 Osaka, Japan

* corresponding author, aleksandra.deptuch@ifj.edu.pl

Supplementary Materials

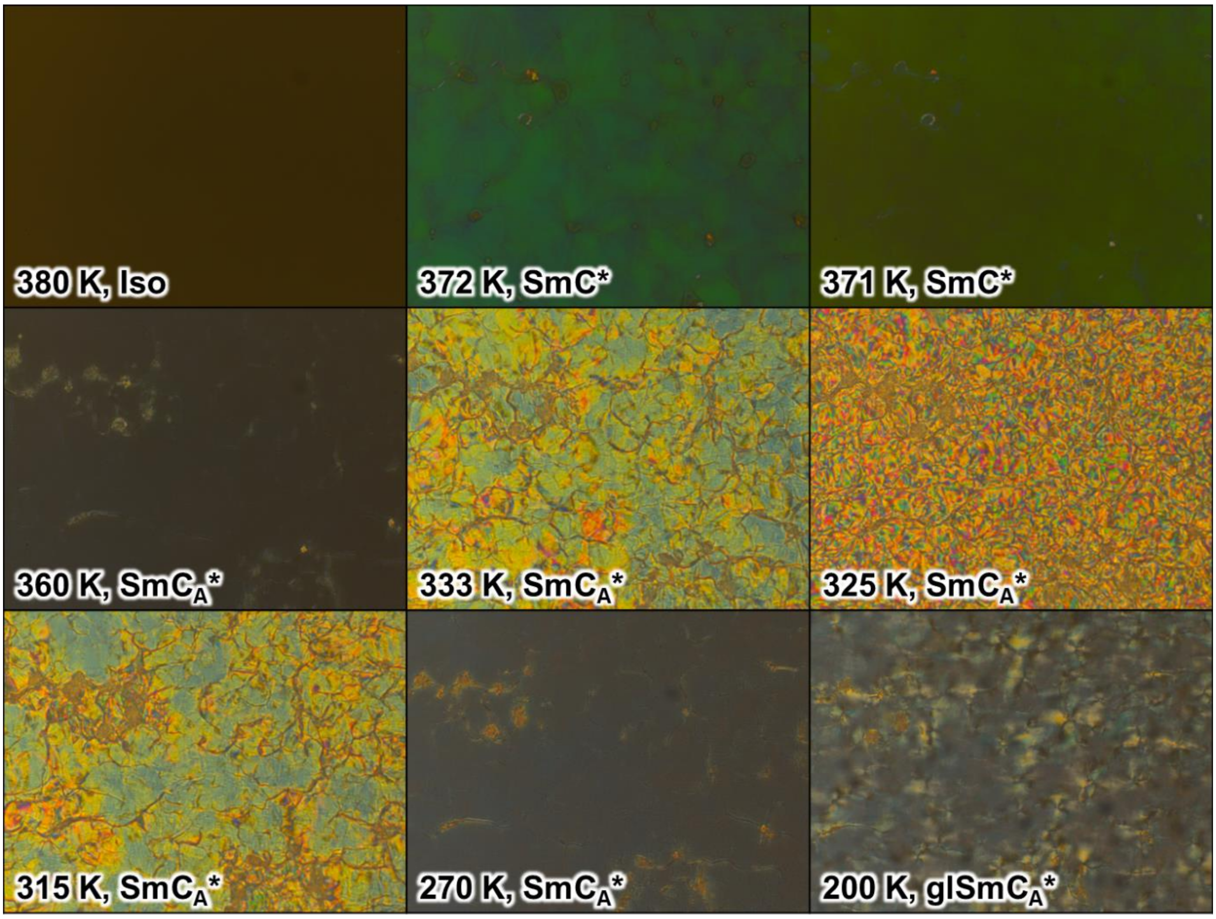

Figure S1. POM textures of 3F5HPhF6 registered on cooling at 10 K/min in the transmission mode. Each image shows an area of 1243 × 933 μm^2.

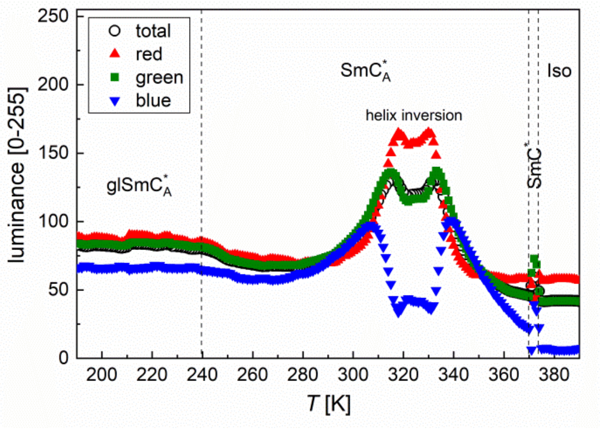

Figure S2. Average weighted luminance and separate contributions of the red, green, and blue components of the POM textures of 3F5HPhF6 obtained on cooling at 10 K/min in the transmission mode.

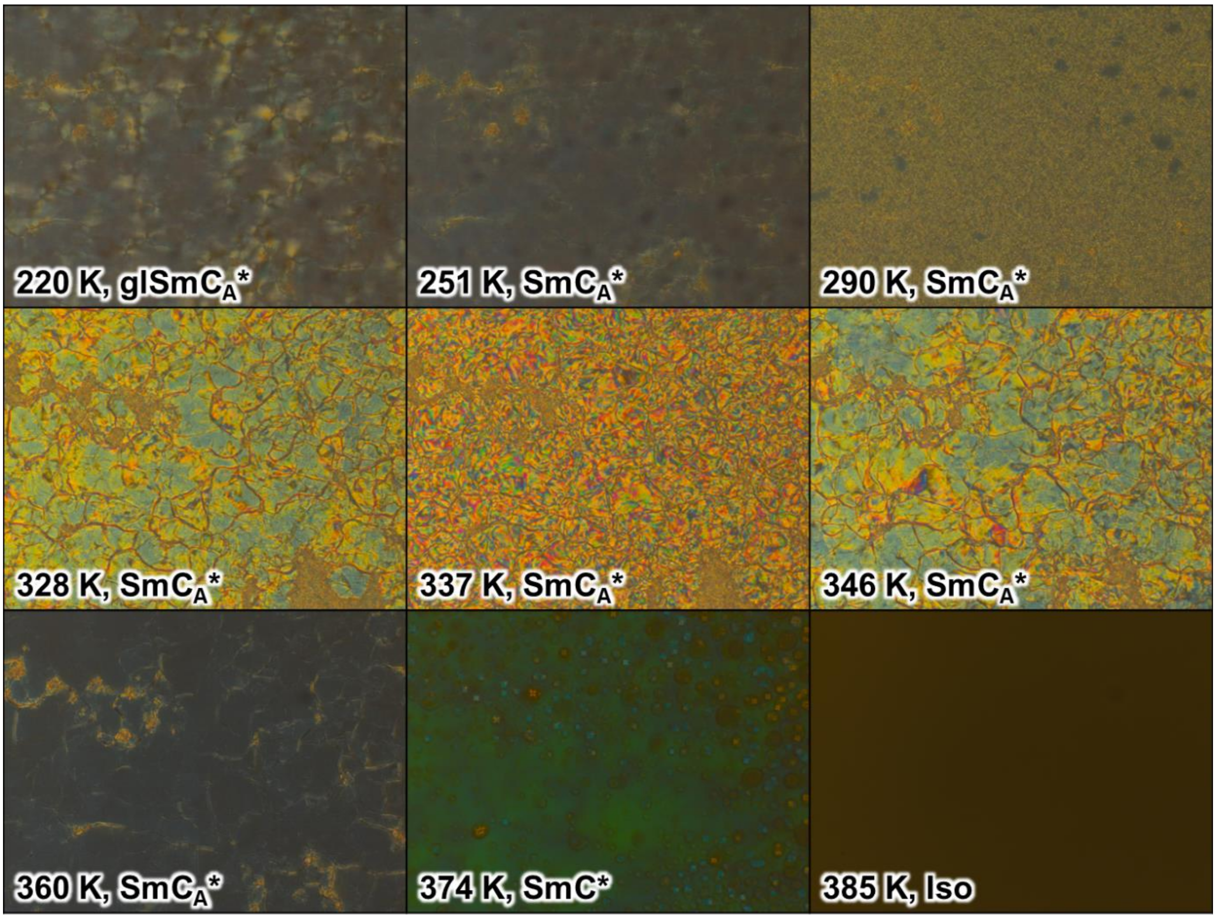

Figure S3. POM textures of 3F5HPhF6 registered on heating at 10 K/min in the transmission mode. Each image shows an area of 1243 × 933 μm^2.

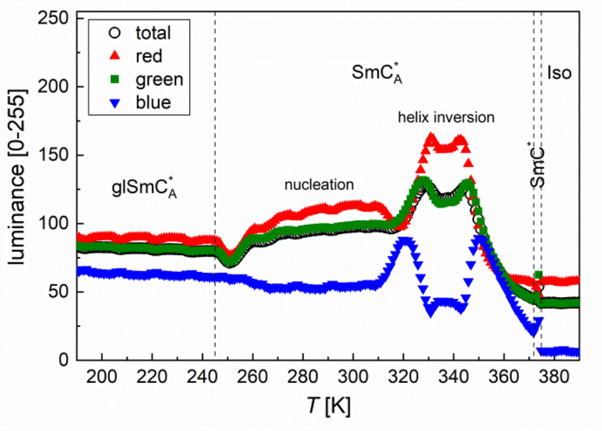

Figure S4. Average weighted luminance and separate contributions of the red, green, and blue components of the POM textures of 3F5HPhF6 obtained on heating at 10 K/min in the transmission mode.

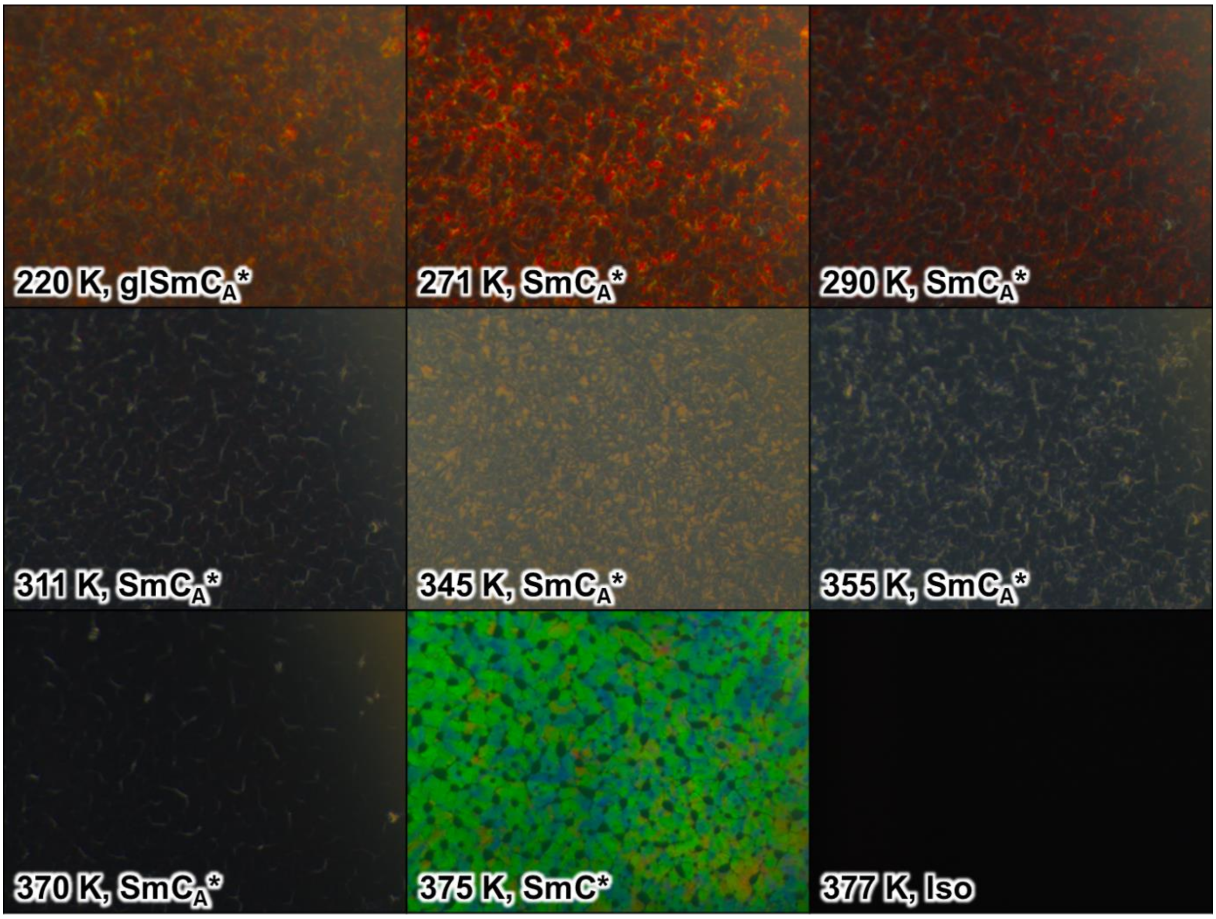

Figure S5. POM textures of 3F5HPhF6 registered on heating at 10 K/min in the reflection mode. Each image shows an area of 622 × 466 μm^2.

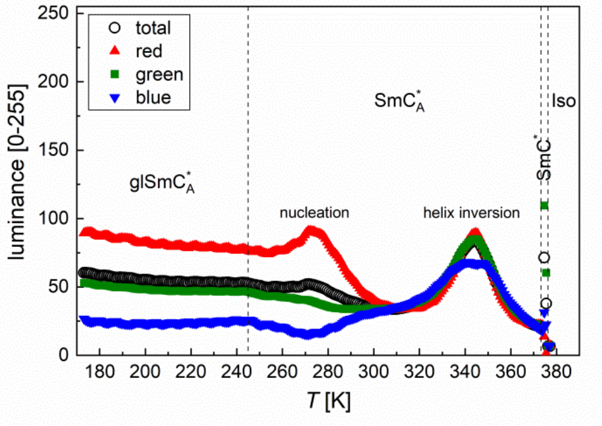

Figure S6. Average weighted luminance and separate contributions of the red, green, and blue components of the POM textures of 3F5HPhF6 obtained on heating at 10 K/min in the reflection mode.

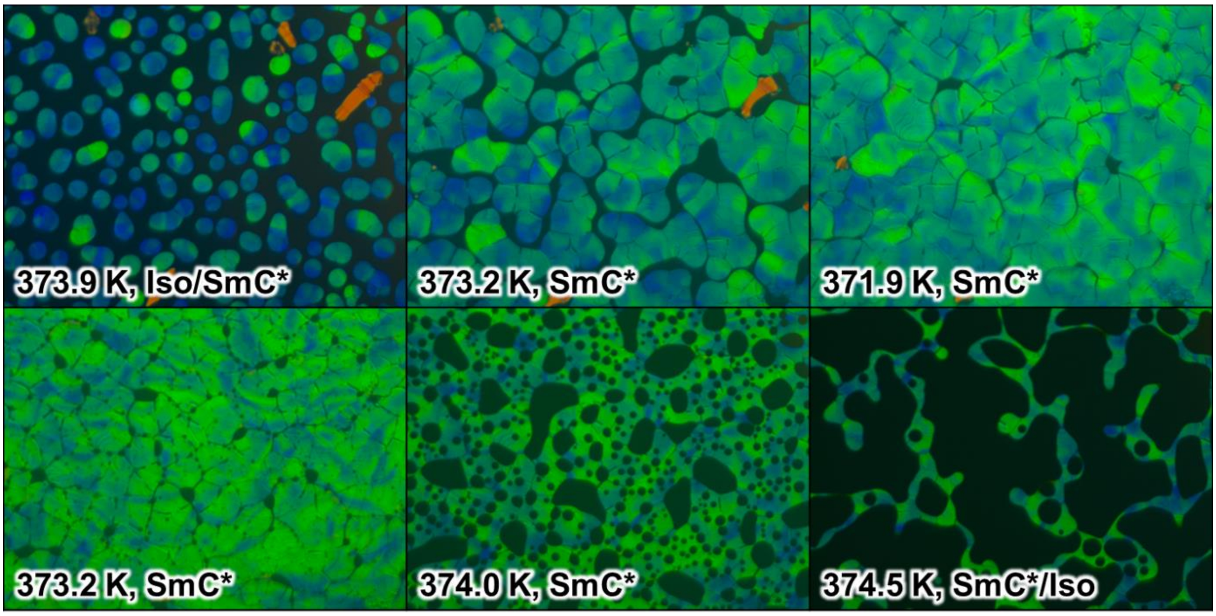

Figure S7. POM textures of 3F5HPhF6 registered on cooling (upper row) and heating (bottom row) at 1 K/min in the reflection mode. Each image shows an area of 622 × 466 μm^2.

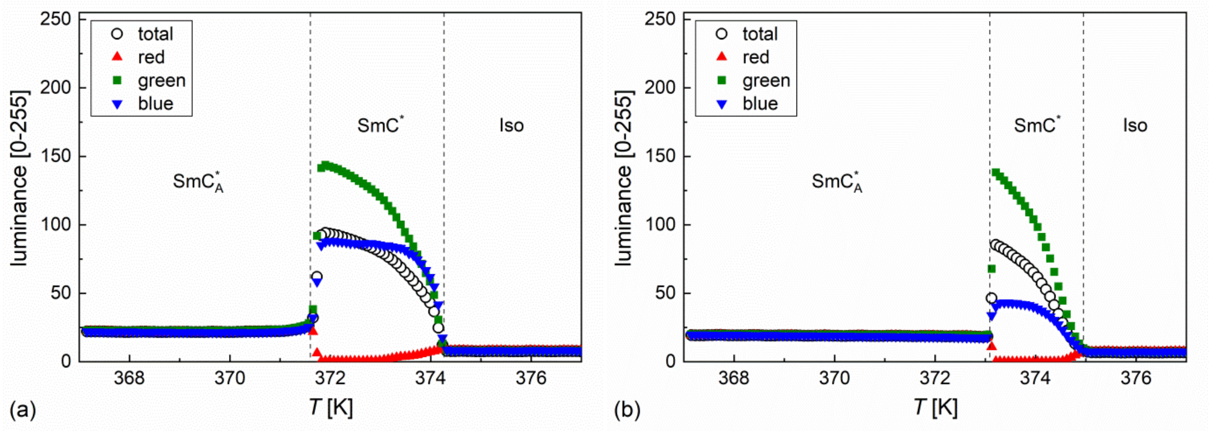

Figure S8. Average weighted luminance and separate contributions of the red, green, and blue components of the POM textures of 3F5HPhF6 obtained on cooling (a) and heating (b) at 1 K/min in the reflection mode.
